# A Testing Method for Small Specimen Bulging Test for Evaluating the Mechanical Property of High-Density Polyethylene

**DOI:** 10.3390/polym17111425

**Published:** 2025-05-22

**Authors:** Bo Zhao, Yunxiang Chen, Lixian Wang, Yuxin Yu, Qiang Dai, Zaixin Li

**Affiliations:** 1 State Key Laboratory of Low-Carbon Thermal Power Generation Technology and Equipments, China Special Equipment Inspection and Research Institute, Beijing 100029, China; wlxhome263@163.com (L.W.); yu13716351246@163.com (Y.Y.); daiq2810@163.com (Q.D.); lizaixin212@163.com (Z.L.); 2 Electric Power Research Institute of State Grid Fujian Electric Power Company Limited, Fuzhou 350007, China

**Keywords:** high-density polyethylene, finite element modeling, hydraulic bulging test, small specimen, mechanical properties

## Abstract

High-density polyethylene (HDPE) is a key material in modern engineering, highly valued for its versatility and wide range of applications. However, accurately assessing its mechanical performance under complex stress conditions has remained a challenge. This study developed an improved small specimen bulging test (SBT) combined with finite element simulation using ABAQUS 2017 software to more precisely evaluate the deformation resistance of HDPE. Both the experimental and simulation results showed that during the hydraulic bulging test, the maximum bulging pressure of the HDPE specimens reached approximately 22 MPa, with a maximum bulging height of about 2.6 mm. The material exhibited three distinct deformation stages: elastic deformation, plastic deformation, and large deformation near failure. Based on the experimental data and simulation results, we established a more accurate material response model for HDPE, which not only better reflects the material’s behavior under actual stress conditions but also provides a reliable basis for engineering design and material selection.

## 1. Introduction

High-density polyethylene (HDPE) has emerged as a cornerstone material in modern engineering, renowned for its versatility and reliability across a broad spectrum of applications. Its use in constructing pressure-bearing infrastructure, including natural gas pipelines, district heating systems, and medical-grade fluid transfer lines, is a direct result of its exceptional mechanical properties, ease of processing, and robust corrosion resistance [1]. The mechanical characteristics such as strength and elastic modulus affect the structural strength and service life of HDPE-based pressure equipment. As such, understanding its failure mechanisms and mechanical behaviors has become a central focus of research in both academic and industrial settings.

In recent years, significant strides have been made in the study of HDPE’s mechanical properties. For example, through advanced spectroscopic techniques, researchers like Reis et al. [2] have demonstrated the effect of strain rate and temperature on the mechanical properties of HDPE through a series of tensile tests. In another study, Briody et al. [3] reported the applicability of the superelastic and viscoelastic constitutive laws. Additionally, Grytten et al. [4] employed digital image correlation methods to precisely measure the surface strains of HDPE samples under various loading conditions, providing valuable experimental data for validating theoretical models.

However, despite these achievements, there are still several critical gaps in our current knowledge. In the realm of testing methods, the uniaxial tensile test remains a widely used standard for determining mechanical properties. While it offers simplicity and convenience, it is deeply rooted in the assumptions of classical material mechanics and fails to mimic the complex stress states that HDPE experiences in real-world applications [5,6]. In practical scenarios, pressure-bearing structures such as pipelines and storage vessels are often subjected to multi-axial and symmetric constraints, placing the HDPE material in a plane-stress state. The mechanical parameters obtained from uniaxial tensile tests, such as Young’s modulus and ultimate tensile strength, can deviate significantly from the values relevant to actual service conditions [7,8]. This discrepancy can lead to inaccurate predictions of material behavior and potential safety risks in engineering designs.

The small punch test (SPT) was developed to address the limitations of uniaxial testing for small specimens under plane-stress conditions. Nevertheless, it is not without its own problems. One of the major challenges is the friction between the specimen and the loading device. This friction can introduce additional forces and moments during the test, complicating the stress distribution within the specimen and making it difficult to accurately interpret the test results. Moreover, numerical analysis of SPT data is often hindered by the complex nature of friction-related effects, which are difficult to model precisely [9,10]. Some alternative methods, like the one proposed by Wang [11], have attempted to overcome these issues but are limited in their applicability. Wang’s method, for instance, is only suitable for metallic materials due to the unique challenges posed by polymers, such as their susceptibility to chemical degradation under certain testing conditions and their sensitivity to over-pressurization [12,13,14].

Regarding constitutive models, HDPE exhibits a highly complex mechanical response that defies simple characterization. The traditional linear elastic constitutive model, based on Young’s modulus and Poisson’s ratio, is inadequate for capturing the nonlinear elastic and plastic behaviors of HDPE, especially during large deformation and post-yielding processes [15]. More advanced constitutive models, such as the Marlow model and the Arruda–Boyce model [16], have been proposed to account for the nonlinearity. However, these models still struggle to incorporate all the factors that influence HDPE’s mechanical behavior. For example, the effects of temperature, strain rate, and material microstructure on HDPE’s mechanical properties are not fully considered in these models. Temperature can significantly alter the viscoelastic properties of HDPE, while strain rate can affect its yield strength and ductility. The complex microstructure of HDPE, including its degree of crystallinity and the distribution of crystallites, also plays a crucial role in determining its mechanical response but is often oversimplified in existing models [17,18].

Against this backdrop, the present study aims to develop an improved testing method, the small specimen bulging test (SBT), for evaluating the mechanical properties of small HDPE specimens. The main advantage of this method is its ability to evaluate the mechanical properties of materials under symmetric constraint conditions. Compared to traditional uniaxial tensile tests, the underlying micromechanisms of deformation under symmetric constraints are different, leading to significant variations in the resulting mechanical performance assessments. This approach more closely simulates the actual service conditions of equipment such as pipelines and pressure vessels, which operate under plane-stress states, thereby enabling a more accurate prediction of material behavior under real-world operating conditions. By carefully selecting appropriate constitutive relations and deformation parameters for fitting and using the finite element simulation software ABAQUS to simulate the experimental process, we seek to establish a more accurate mechanical response model for HDPE. This model will not only better capture the material’s behavior under realistic stress conditions but also provide a more reliable basis for engineering design and material selection, thereby filling in the existing scientific gaps and addressing the practical needs in the field.

## 2. Experimental Section

### 2.1. Specimen Preparation

In accordance with the ASTM F 2183-02 standards [19], we prepared high-density polyethylene (HDPE) specimens for the experiments. The HDPE raw material used was M7600 supplied by SINOPEC (Beijing, China). Specimens were produced via a bar-extruded process at 165 °C. The HDPE material was first processed into 1 mm thick sheets. Then, using a 10 mm diameter punch, circular disk specimens with a diameter of approximately 10 mm and a thickness of approximately 1.0 mm were carefully cut from the sheets to ensure accurate dimensions. After cutting, the edges of the specimens were polished to achieve a smooth surface finish. Surface roughness was measured and recorded to ensure that it fell within the specified range. A total of three specimens were prepared in this manner to ensure the statistical significance of the experimental results.

### 2.2. Experimental Setup

**Figure 1:**
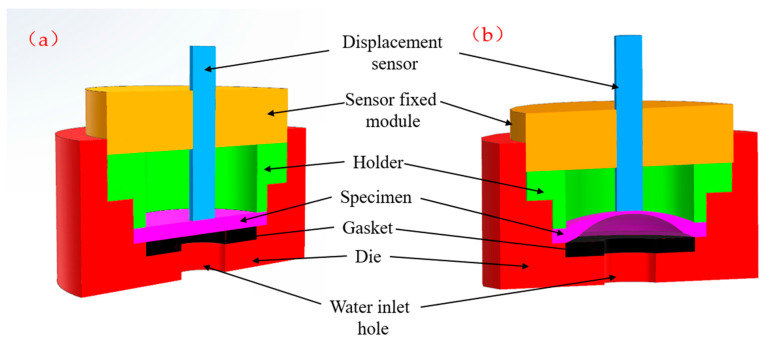

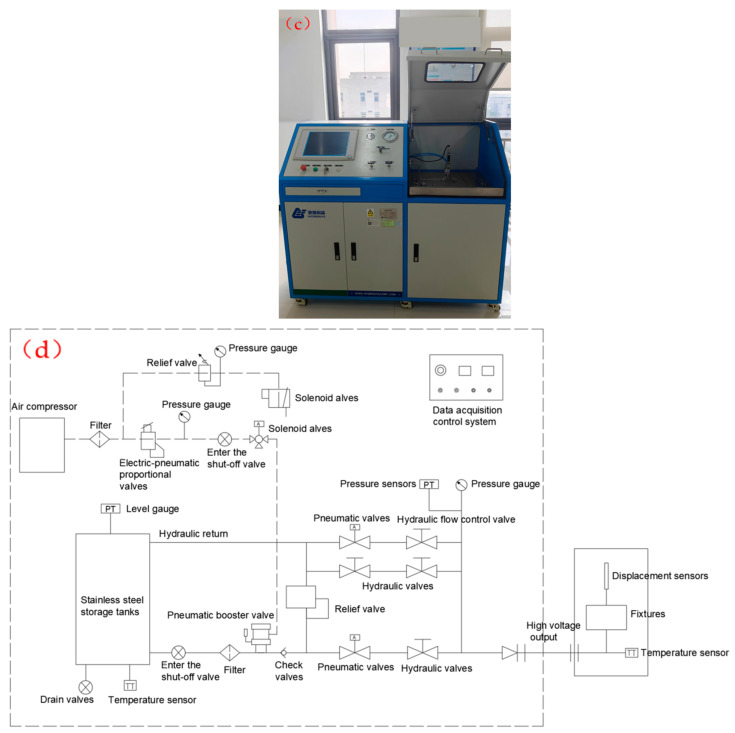
The situation of testing device at (**a**) specimen placed, (**b**) specimen bulging test, (**c**) photograph of the testing device, and (**d**) schematic diagram of the pressurization device.

The experimental setup consisted of a hydraulic system, a high-precision pressure sensor, a high-accuracy displacement sensor, and a data-acquisition system. The hydraulic system was used to apply pressure to the specimens. Figure 1 presents the experimental device, clearly showing its key components and how it is connected to the rest of the experimental setup. The machine is self-developed. The test parameters are set as follows: initial pressure of 0.5 MPa, test temperature at room temperature, and pressure increase rate of 0.2 MPa/s. The high-precision pressure sensor, with an accuracy of ±0.05 MPa and a working range up to 150 MPa, was installed in the hydraulic line to measure the pressure applied to the specimen precisely. The high-accuracy displacement sensor, with a resolution of 0.001 mm and a range of 7.5 mm, was positioned to measure the displacement of the specimen at the center point during the test. Additionally, a pressure pump capable of providing a maximum pressure of 25 MPa with an accuracy of ±0.05 MPa was used. The data-acquisition system, with a sampling rate of 200 Hz, was used to collect the data from the pressure and displacement sensors in real time.

### 2.3. Experimental Procedure

Before the test, each specimen was carefully placed in the testing apparatus, ensuring proper alignment and secure fixation. The holder and die, made of steel and aluminum alloy, respectively, were designed to provide the necessary support and confinement for the specimen during the hydraulic bulging process. The contact between the specimen and the holder/die was modeled as a contact-pair with appropriate friction coefficients in the subsequent finite element method (FEM) simulations.

During the test, the hydraulic pressure gradually increased at a controlled rate. The pressure-increasing rate was set based on preliminary tests, ensuring that the deformation process of the specimen was quasi-static. The pressure–displacement data were continuously recorded by the data-acquisition system throughout the test until the specimen was fractured. The fracture area of the specimens was consistently located at the center, indicating a relatively uniform stress distribution on the surface and a tendency towards a state of minimum surface strain.

### 2.4. FEM Model Integration

A detailed FEM model should be determined by the experimental results, including FEM mesh, boundary conditions, and a description of the used settings and elements. The FEM model scheme is as follows.

Boundary Conditions

Displacement Boundary Conditions:

At the bottom surface of the holder, all degrees of freedom (translational in the *x*, *y*, and *z* directions and rotational around the *x*, *y*, and *z* axes) are constrained, as shown in Equations (1) and (2).

(1) $$u_{x}=u_{y}=u_{z}=0$$

(2) $$\theta_{x}=\theta_{y}=\theta_{z}=0$$

where *u* is the displacement and *θ* is the angle of rotation.

This mimics the fixed-base condition in the actual experimental setup where the holder is firmly mounted.

For the die, the outer-most surface that is not in contact with the specimen is also fixed in all degrees of freedom, ensuring its stability during the simulation and enabling it to apply the required pressure on the specimen.

Pressure Boundary Conditions:

A uniformly distributed pressure load, equivalent to the hydraulic pressure in the experiment, is applied on the inner surface of the die that contacts the HDPE specimen. The pressure value is based on the experimental data and gradually increased during the simulation to replicate the loading process.

Supports:

The holder acts as the main support for the specimen. In the FEM model, its support function is represented by the fixed-displacement boundary conditions at its bottom surface.

Small support pads are assumed between the specimen and the holder at the specimen’s edges. In the FEM model, the interaction between the specimen and these support pads is modeled as a contact-pair with a high-friction coefficient to prevent relative motion.

Load Application:

The load on the HDPE specimen is the hydraulic pressure transmitted through the die. It is applied step-by-step, with each step corresponding to a specific time increment in the simulation.

The pressure load direction is always normal to the inner surface of the die in contact with the specimen, consistent with the physical hydraulic bulging process. A small random variation in the pressure distribution, based on experimental measurements of pressure uniformity, is introduced to make the simulation more realistic.

### 2.5. Experimental Data Analysis

The recorded pressure–displacement curves were analyzed in detail. We calculated various parameters from these curves, such as the initial elastic modulus, yield point, and ultimate strength of the HDPE specimens. The thickness reduction–pressure curve was also obtained and found to be fitted well by an exponential equation. This finding not only validates the feasibility of the numerical analysis but also provides valuable insights into the material’s behavior under loading.

To ensure the reliability of the experimental results, we conducted a comprehensive statistical analysis. For each measured parameter, we calculated the minimum, maximum, median, mean, range, standard deviation, and measurement uncertainty. The results of this statistical analysis were presented in a table, which clearly shows the distribution and variability of the data.

### 2.6. Comparison with FEM Simulations

The experimental data were compared with the results obtained from the FEM simulations. Both the pressure–displacement curves from the experiments and simulations exhibited a rise–fall–rise process, indicating that the FEM simulations could adequately describe the experimental data. However, some minor differences were also observed, which were further analyzed in the Section 4. These differences were mainly attributed to factors such as the simplification of the FEM model, the assumptions in the material constitutive model, and the manufacturing tolerances of the specimens.

## 3. Results

### 3.1. Test Results

**Figure 2:**
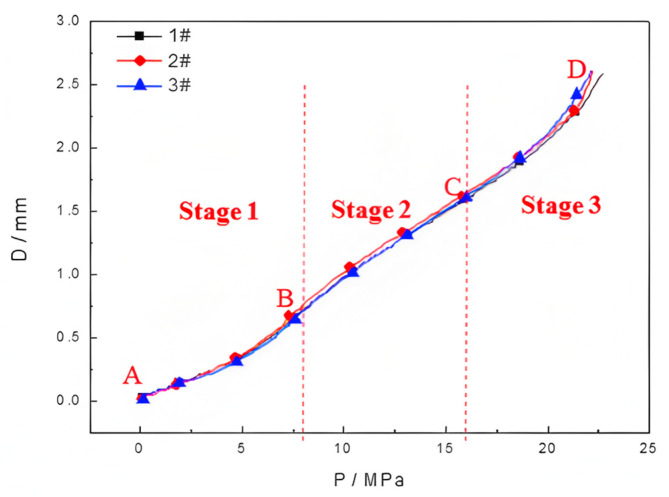
The pressure–displacement curves of the three sets of the HDPE samples.

Figure 2 shows the pressure–displacement curves of the three sets of the HDPE samples during the hydraulic bulging test. As can be observed, the concavity and convexity of the pressure–displacement curves change during the deformation of HDPE by hydraulic bulging. Based on the definition of concavity and convexity, the parameter Ꞁ can be determined as shown in Equation (3).

(3) $$Ꞁ=f^{\prime \prime }(U)=\frac{d^{2}P}{dU^{2}}=\frac{d^{2}(dP/dU)}{dU}$$

where *P* represents pressure and *U* represents strain energy, which is used to evaluate the degree of damage of the material and is often used in the analysis related to material damage. The starting point of the pressure–displacement curve and inflection point at Ꞁ = 0 are selected, followed by the calibration of the four points (A, B, C, and D) as per the time evolution law. In other words, the pressure–displacement curve is divided into three stages, stage 1 (A–B section), stage 2 (B–C section), and stage 3 (C–D section), as shown in Figure 2. Moreover, stage 1, stage 2, and stage 3 in Figure 2 represent the elastic deformation stage, plastic deformation stage, and large deformation stage approaching fracture of the material, respectively.

The sample thickness T (mm), swelling limit pressure P_max_ (MPa), maximum bulging height D_max_ (mm), maximum deviation of bulging pressure P_δ**max**_ (%, relative to sample #1), and maximum deviation of bulging height D_δmax_ (%, relative to sample #1) for the three samples were calculated, as shown in Table 1.

As per Figure 2 and Table 1, the maximum deviation of the pressure and bulging height of specimens 2 and 3 relative to sample 1 is 2.64% and 0.77%, respectively. The obtained results exhibit an optimal degree of coincidence of the three curves, thus confirming the repeatability and reliability of the method.

### 3.2. Morphological Observation

After the removal of the pressure, the macro-morphology of the samples, which were loaded to four distinct stages, as illustrated in Figure 2, was examined using a KH-8700 3D stereoscopic microscope (manufactured by Hirox Co., Ltd., Tokyo, Japan). As shown in Figure 2, the applied pressure at the midpoint of stage 1 (A–B section) is 4 MPa, whereas it is 12 MPa and 20 MPa at stage 2 (B–C section) and stage 3 (C–D section), respectively. Finally, the critical/maximal pressure is 22 MPa at the point of fracture.

**Figure 3:**
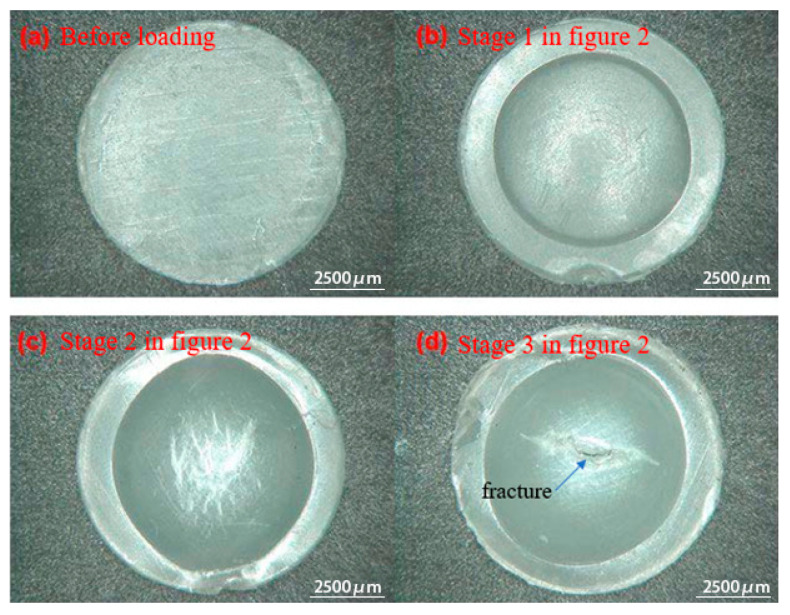
The macro-morphology of the samples at different stages after the SBT.

**Figure 4:**
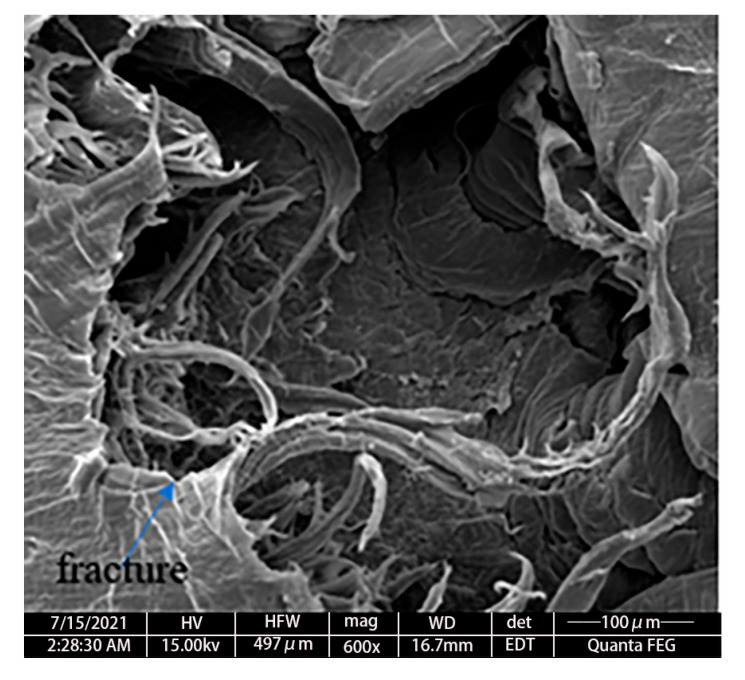
The micro-morphology of the sample after hydraulic bulging.

As can be observed from Figure 3a, when loaded to stage 1 (A–B section), the surface morphology of the sample is similar to that before loading and remains flat. This indicates that the sample is in the elastic deformation stage at this time and can return to its original state after unloading. In Figure 3b, the sample shows a prominent spherical crown. Although the surface of the sample remains smooth, the crazes that like defects that occur in polymer materials under stress are visible to the naked eye. Therefore, the material in stage 2 (B–C section) enters the yield state; thus, the bulging deformation cannot be recovered. In Figure 3c, an obvious crazing is observed on the top of the sample, thus indicating a large extent of damage accumulated inside the sample in stage 3 (C–D section), along with the initiation of the macroscopic defects. In Figure 3d, obvious cracks are observed on the top of the sample, thus demonstrating that the maximum amount of deformation and thinning of the sample occur at the top, eventually leading to sample fracture. The micro-morphology of the cracked sample in Figure 3d is demonstrated in Figure 4.

Microscopic cracks are observed on the stress-deformed surfaces of the sample after bulging and cracking, as shown in Figure 4. The images were obtained using a Quanta FEG scanning electron microscope (manufactured by Thermo Fisher Scientific, Waltham, MA, USA). This indicates that due to hydraulic pressure, the stress on the surface of the specimen is uniform, and the sample tends to attain the strain state with the smallest surface area. Thus, the sample is analyzable and can be deduced by the mathematical function.

### 3.3. Establishment of Constitutive Model

According to previous studies [15], HDPE exhibits obvious nonlinear characteristics in the elastic deformation stage; thus, the conventional linear elastic constitutive model based on Young’s modulus and Poisson’s ratio cannot describe the mechanical behavior of HDPE. After comparing the various models, the Marlow constitutive model [16] was selected to characterize the elastic part of the stress–strain relationship in this study, and its strain potential energy function can be given as Equation (4).

(4) $$U=U_{dev}(I_{1})+U_{vol}(J_{el})$$

where *U*_dev_ and *U*_vol_ are the deviator and volume of the strain energy function, respectively, *J_el_* is the elastic volume ratio, and *I*_1_ is the first deviatoric strain invariant.

It should be noted that the Marlow constitutive model cannot accurately describe the mechanical response of the material after the yielding phenomenon [17,18]. Therefore, the isotropic plastic theory was introduced in this study to modify the plastic strain by using Equation (5), and the isotropic power law hardening $\bar{\sigma }=k{\left(\varepsilon_{0}+\bar{\varepsilon }\right)}^{N}$ was used for fitting. k is 39.85, ε_0_ is 0.135, and N is 0.117.

(5) $$\varepsilon^{\rho }=\varepsilon -\varepsilon^{e}=\varepsilon -\varepsilon_{y}-\frac{\Delta \sigma }{E}$$

where *ε_y_* is the total strain before yielding, *E* is the tangent modulus of the yield point, and $\Delta \sigma =\sigma -\sigma_{y}$ represents the difference between the true stress and yield stress *σ_y_*.

In addition, in order to account for the stiffness degradation caused by the damage, the ductile damage theory [20,21] was further introduced in the constitutive model. The stiffness degradation associated with the active failure mechanism was evaluated using a scalar damage variable *D*. At any given time during the numerical analysis, the stress tensor in the material is given by the following scalar damage equation:

(6) $$\sigma =(1-D)\overset{―}{\sigma }$$

where σ is the effective stress tensor calculated based on the current increment, referred to as the increment in the current loading step, and $\overset{―}{\sigma }$ refers to the stress tensor obtained without considering the material damage. The value of the scalar damage variable *D* lies between 0 and 1. At *D* = 1, the material loses its bearing capacity. The damage evolution after failure is defined by the energy criterion provided by the ABAQUS 2017 software, and the fracture is simulated by the energy required for failure (fracture energy) after the damage initiation [22,23].

### 3.4. Finite Element Simulation

**Figure 5:**
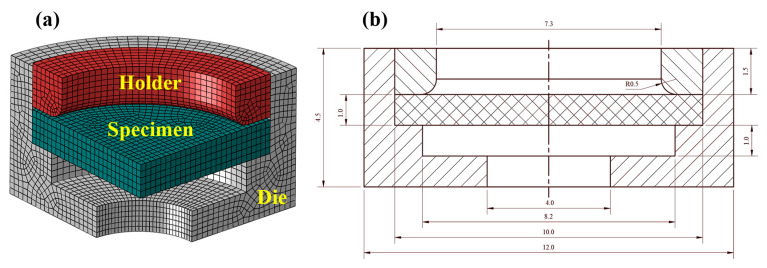
The finite element model of the hydraulic bulging experiment: (**a**) 3D model, (**b**) section view drawing.

The finite element model of the hydraulic bulging experiment is shown in Figure 5a. The thickness of the sample is consistent with the experiment (set to a nominal thickness of 1 mm). The bulging experimental model represents an axisymmetric model. After considering the speed of the numerical simulation and accuracy of the results, the whole sample was segmented, and the 1/4 model was used for the calculation in this study. In order to be consistent with the experimental loading conditions, the finite element model was divided into three components: holder, specimen, and die. The number of elements for species, holder, and die are 2700, 7530, and 2430. The dimensions of the cross-section are presented in Figure 5b.

Considering that the specimen is subjected to the bending load during the loading process, a three-dimensional eight-node reduced integral element (C3D8R) has been used for meshing to avoid shear self-locking. On introducing the concept of hourglass stiffness in the reduced integral element in ABAQUS, a reasonable mesh refinement can effectively improve the accuracy of the numerical calculation. Therefore, five layers are divided along the thickness direction of the specimen to control the error [24].

Further, the explicit integral ABAQUS/Explicit is used in the calculation, and the model mass is scaled to 10. With respect to the materials, the holder and die are set as the iron-based metal materials, with an elastic modulus of 200 GPa, a Poisson’s ratio of 0.3, and a density of 7.9 kg/mm^3^. As the stiffness of the holder and die is much higher than that of the HDPE material, the isotropic linear elastic constitutive model is used to describe these materials.

**Figure 6:**
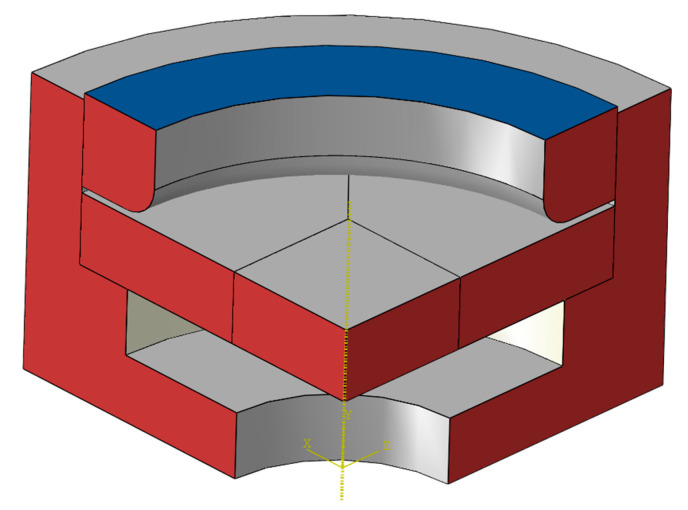
The schematic diagram of the loading in the hydraulic bulging simulation.

The loading in the numerical calculation is consistent with the experiment, which is divided into two steps. In the first step, 30 MPa is applied to the blue surface in Figure 6 to ensure that there is no unexpected relative sliding between the fixture and the specimen. In the second step, the pressure control scheme is adopted, and the lower surface of the specimen is pressurized at a constant rate of 0.2 MPa/min until fracture occurs. In terms of constraints, the bottom surface of the fixture is set as the fixed end to constrain all degrees of freedom. At the same time, the two symmetrical planes (the red surface in Figure 6) are subjected to the symmetric constraint along the circumference.

A large static friction is observed between the HDPE sheet and fixture as the specimen and fixture are fixed by the down pressure load of a compression ring. In the simulation, the friction contact with the fixture is set on the upper and lower surfaces of the specimen, and the standard Coulomb friction definition is used. Based on previous studies [25], the friction coefficient value is set as 0.5.

**Figure 7:**
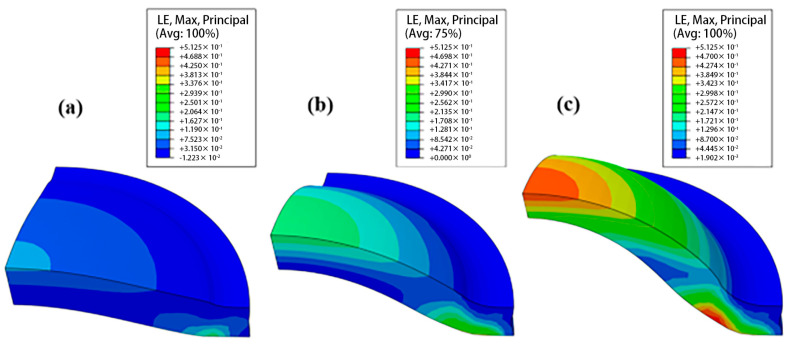
The logarithmic strain nephograms under pressure loads of (**a**) 6 MPa, (**b**) 14 MPa, and (**c**) 21 MPa.

Figure 7 shows the logarithmic strain nephograms of the specimen as a function of the applied pressure during the bulging experiment. In Figure 7, the color gradient represents different levels of logarithmic strain, with warmer colors indicating higher strain values and cooler colors indicating lower strain values. This figure provides insights into the strain distribution characteristics of the HDPE material under the test, which is crucial for understanding its mechanical behavior during the deformation process. Figure 7a–c correspond to pressure loads of 6 MPa, 14 MPa, and 21 MPa, respectively. Two large deformation areas are obvious in the specimen. The first area is located on the opposite side of the contact between the specimen bottom and fixture. This position is mainly affected by the tensile force and gradually produces a plastic flow on loading [26]. The other position is at the top of the sample, which is consistent with the macro-morphology of the HDPE sample after the hydraulic bulging test (as shown in Figure 3).

## 4. Discussion

In this paper, the overestimation of the early-stage displacement growth rate is mainly due to the simplification of the finite element model, like treating HDPE’s non-uniform microstructure as homogeneous, and assumptions in the Marlow constitutive model, which fails to capture the complex elastic–plastic transition at the start of loading. This overestimation distorts the analysis of the experimental results, such as miscalculating the modulus of elasticity, and undermines the accuracy of the conclusions, potentially misguiding product safety assessment and future research. To rectify this, we propose model refinement by incorporating detailed micro-structural information and adjusting mesh density, modifying the constitutive model by adding parameters or coupling with other models, and conducting extensive calibration and validation using diverse experimental data and in situ microscopy techniques.

### 4.1. Experimental Method and Analysis of Deformation Mechanism

**Figure 8:**
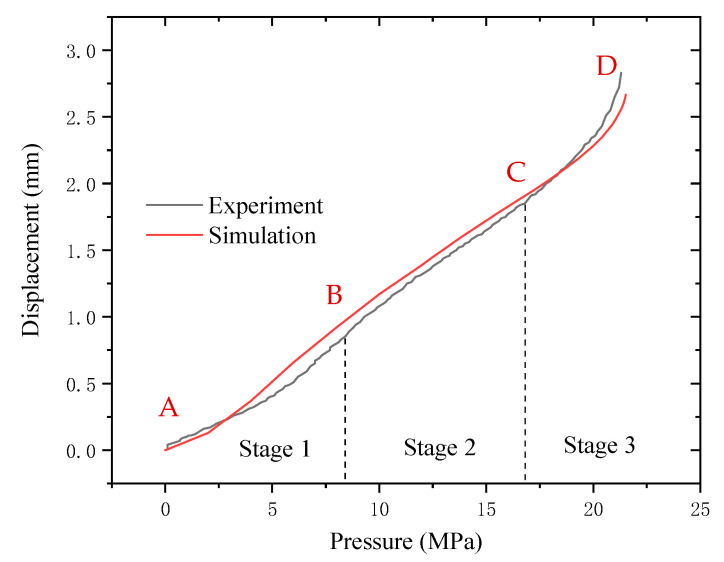
The pressure–displacement curves of the hydraulic bulging of HDPE obtained by the finite element simulation and the experimental results.

Figure 8 shows the pressure–displacement curves of the HDPE sample obtained by the finite element simulation and corresponding experimental results. As can be observed, both methodologies exhibit the same trend prior to failure. Based on the definition of the parameter Ꞁ determined by Equation (3), Ꞁ goes through the process of rising–descending–rising in both curves. This indicates that the finite element simulation can describe the experimental data well, along with revealing the characteristics of the above stages. The main error between the simulation and experimental results is that the finite element method overestimates the top displacement growth rate during the initial loading stage. The predicted value is slightly higher than the experimental data at the end of stage 1 for the different pressurization conditions. However, the observed error is less than 7% and has been effectively corrected in the subsequent analysis. Therefore, the simulation results obtained in this study can be confirmed to be accurate.

Based on Figure 3 and Figure 4, the behavior of the pressure–displacement curve obtained from the hydraulic bulging test can be analyzed as follows:

In stage 1 (A–B section), owing to the initial loading stage, the bulging height and Ꞁ rise rapidly with pressure, thus indicating that in the initial stage of stage 1 (A–B section), the bulge height increases slowly with the increase in pressure and the growth rate accelerates in the later stage. In stage 1 (A–B section), the deformation of the material basically conforms to Hooke’s law [27,28]. Therefore, the material exhibits a linear elastic deformation at this stage. The deformation mechanism is mainly the internal rotation of the flexible macromolecular chain, leading to the reduced chain motion and conformational number [29,30].

In stage 2 (B–C section), the displacement of the sample is linear as a function of pressure. However, at this stage, Ꞁ is lower than stage 1, and the bulging height of the material gradually slows down on increasing the pressure. Thus, the deformation of the material cannot be recovered, thus indicating an increment in the modulus of the material, with the sample entering the plastic deformation zone. In addition to the movement of the chain segments, the deformation mechanism of the sample also includes the orientation change, slip, lamellae fracture, and recrystallization in the crystalline region [31,32].

In stage 3 (C–D section), the degree of bulging of the sample no longer conforms to the linear relationship with pressure, and Ꞁ is observed to increase with pressure. The sample exhibits an obvious crazing morphology, and the macromolecular chains are highly oriented with stress direction. Thus, a high degree of plastic and viscoelastic deformation appears inside the sample [33]. As the craze modulus is lower than that of HDPE, the deformation is mainly concentrated in the craze area. As the bulging stress increases, the sample undergoes a crazing–crack transition. It should be noted that the delamination debonding behavior of the crack position and non-obvious microfibers in Figure 4 are related to the high constraint of the plane-stress state on the deformation of the sample.

**Figure 9:**
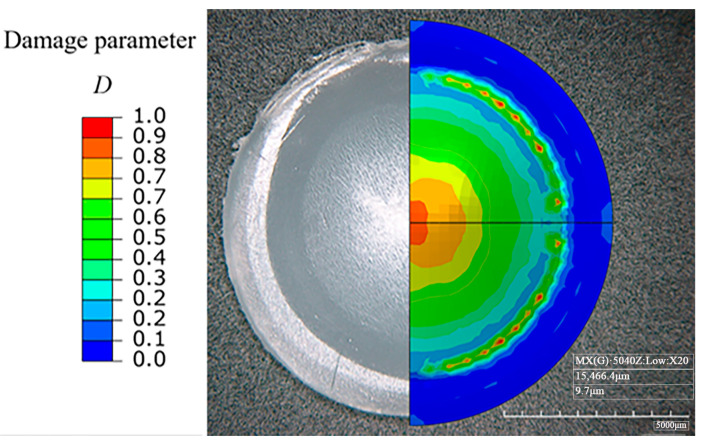
The damage distribution nephogram of the hydraulically bulging specimen.

Figure 9 shows the damage distribution nephogram of the bulging specimen prior to fracture. As can be observed, the position of the maximum damage is located at the top of the specimen and expands in an annular shape, which is consistent with the test results. As compared with other locations, the large deformation area observed during the experiment, especially the white area where the color changes in Figure 9, has a significantly high damage value *D* (between 0.7 and 1).

As the fixing and sealing of the sample and fixture mainly rely on friction, the sample will inevitably slide relative to the fixture during the bulging test, especially near the filet and cavity, thus leading to a deviation in the process analysis. In addition, Figure 9 shows a certain degree of damage at the contact position of the blank holder. In order to prevent the non-top fracture during the experiment, particular attention must be paid to the blank holder of the fixture.

### 4.2. Analysis of Sample Thinning

**Figure 10:**
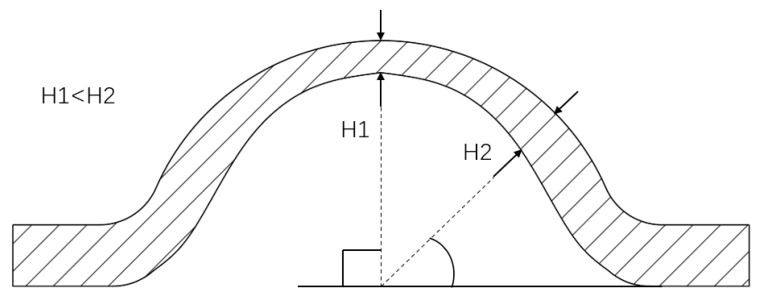
The schematic diagram of the local thinning of the sample.

In the experiment, an obvious local thinning was observed at the top of the hydraulically bulging sample, i.e., the top thickness *H*_1_ was significantly smaller than the side thickness *H*_2_, as shown in Figure 10.

The thinning process forms the basis for the bulging and rupture of the samples. To effectively analyze the process, *H*_1_ and *H*_2_ can be defined as the thicknesses of the specimen at a distance of 0 mm and 2 mm from the center point, respectively, and the thinning thickness *DH* can be defined as follows:

(7) $$DH=H_{2}-H_{1}$$

where *DH* is the thinning thickness, mm; *H*_1_ is the thickness at 0 mm from the center point, mm; and *H*_2_ is the thickness at 2 mm from the center point, mm.

**Figure 11:**
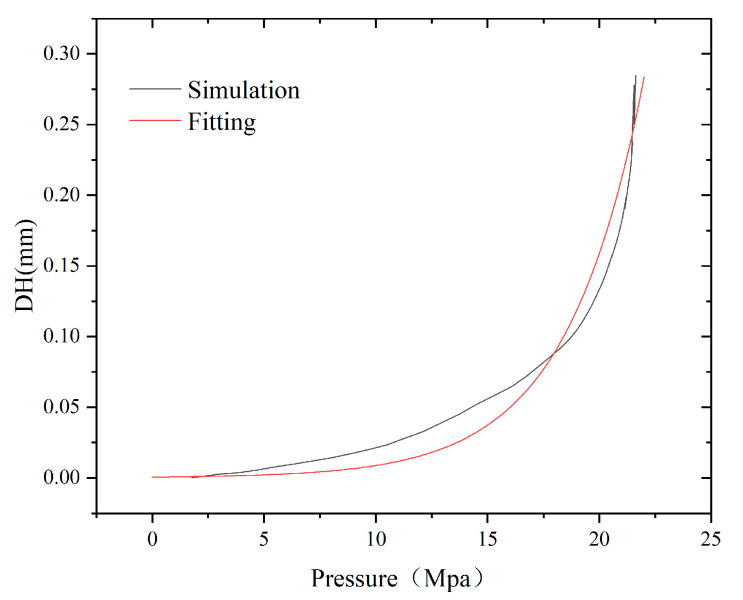
The schematic diagram of local thinning of the specimen.

Figure 11 shows the variation in the thinning thickness as a function of load based on the finite element simulation results. The results exhibit a nonlinear relationship between *DH* and applied pressure. At the early stage of loading, the specimen sheet is in the elastic deformation stage. At this stage, the degree of thinning is less, thus indicating that the specimen is bulging nearly uniformly. As the pressure reaches 10 MPa, the thinning thickness shows an upward trend, resulting from the large plastic deformation of the local material during the middle and late loading stages [34].

Based on the finite element calculation results, the exponential equation (Equation (8)) can be used to fit the relationship between the pressure and thinning thickness, as follows:

(8) DH=Exp(A+Bρ)

where *DH* is the thinning thickness, mm; *ρ* is the loading pressure, bar.; and *A* and *B* are the correction coefficients for the curves in Figure 11, where A is −7.64 and B is 0.29.

In our study on the mechanical properties of high-density polyethylene (HDPE) using a numerical model, the influence of mesh size on the model’s results is a crucial aspect. By varying the mesh size in our simulations, we were able to observe distinct trends. When a coarse mesh was employed, computational efficiency increased, yet the precision of the outcomes was compromised. For example, in the hydraulic bulging test simulation involving HDPE specimens, a coarse mesh inadequately resolved the stress and strain distributions in key regions, resulting in discrepancies in the anticipated deformation profiles. On the other hand, a fine mesh provided more accurate results, closely resembling the experimental observations, but it significantly increased computational time. Regarding the multi-level Monte Carlo simulation method, we explored its application in reliability analysis. This method is based on generating multiple levels of approximations of the numerical model, each with a different mesh size [35]. By doing so, it can efficiently estimate the variance and uncertainty in the model’s output. In the context of our HDPE research, this method can be used to assess the reliability of our numerical model in predicting the mechanical properties of HDPE under different conditions. For example, it can help us determine the probability of the failure of HDPE in pressure-bearing applications by considering the uncertainties associated with the mesh size and other model parameters. This not only enhances the reliability evaluation of our numerical model but also broadens the scope of our research, allowing for a more comprehensive understanding of the behavior of HDPE in real-world scenarios. We have successfully tested and analyzed the mechanical properties of HDPE materials under symmetric constraints using the hydraulic bulging method. Next, we will optimize the fixture structure and specimen size and explore the effectiveness of this method in testing various flexible non-metallic materials.

## 5. Conclusions

This research investigated the mechanical properties of HDPE through the development of the SBT and constitutive modeling. The SBT addresses the limitations of traditional uniaxial tensile tests by simulating multi-axial stress conditions, which are representative of real-world applications such as high-pressure pipelines or storage vessels. This approach enables the evaluation of HDPE’s mechanical performance under complex stress states. The constitutive model developed in this study incorporates strain-rate sensitivity and temperature-dependent behavior, allowing for predictions of HDPE’s mechanical response under various operating conditions. Numerical simulations using ABAQUS were conducted to create finite element models that included HDPE’s material properties, specimen geometries, and boundary conditions. These simulations predicted deformation patterns, stress distributions, and failure mechanisms during the SBT. Experimental validation was performed on HDPE specimens using measurement techniques to record key data. The comparison between numerical predictions and experimental results showed strong agreement. The pressure–displacement curves from the simulations matched closely with the experimental data, and the strain distribution maps also demonstrated consistency. These findings provide insights into HDPE’s mechanical behavior under realistic stress conditions, which can inform the design and safety assessment of HDPE-based infrastructures. Future research could focus on investigating HDPE’s long-term creep behavior under cyclic loading and the effects of different manufacturing processes on its mechanical properties. Such studies could contribute to further improvements in material performance and infrastructure durability.

## Figures and Tables

**Table 1:** The data acquired from the pressure–displacement curves of the three sets of the HDPE samples.

	T/mm	P_max_/MPa	D_max_/mm	P_δmax_/%	D_δmax_/%
1	1.09	22.7	2.59	0	0
2	1.09	22.2	2.61	2.2	0.77
3	1.06	22.1	2.61	2.64	0.77

## Data Availability

The original contributions presented in this study are included in the article. Further inquiries can be directed to the corresponding author(s).

## Abbreviations

The following abbreviations are used in this manuscript:

Term	Definition
HDPE	High-density polyethylene
SBT	Specimen bulging test
FEM	Finite element method
MPa	Megapascal (unit of pressure)
GPa	Gigapascal (unit of pressure)
Pa	Pascal (unit of pressure)
*u_x_*, *u_y_*, *u_z_*	Displacements in the x, y, and z directions, respectively
*θ_x_*, *θ_y_*, *θ_z_*	Rotational angles around the x, y, and z axes, respectively
C3D8R	A type of finite element, linear hexahedral reduced-integration element
